# Evolution of Root Morphology in Table Beet: Historical and Iconographic

**DOI:** 10.3389/fpls.2021.689926

**Published:** 2021-08-10

**Authors:** Irwin L. Goldman, Jules Janick

**Affiliations:** ^1^Department of Horticulture, University of Wisconsin-Madison, Madison, WI, United States; ^2^Department of Horticulture and Landscape Architecture, Purdue University, West Lafayette, IN, United States

**Keywords:** beet, *Beta vulgaris*, table beet, morphology, vegetable evolution

## Abstract

The *Beta vulgaris* complex includes sugar beet, mangel wurzel, Swiss chard, fodder beet, and table beet. Mangel wurzel and fodder beet are considered to be the same general crop type, with the former possessing lower dry matter content (<13%) than the latter. Mangel is likely derived from crosses between table beet and chard, while fodder beet may have a more recent origin, arising from crosses between mangel and sugarbeet. The table beet was derived from the wild sea beet, *B. vulgaris* (L.) subsp. *maritima* (L.) Arcang, with small non-spherical roots. Table beet is presently a popular vegetable cultivated for its pigmented roots, typically red but also yellow and other colors. Wild forms were consumed in antiquity mainly for their leaves with roots used medicinally. Beet is referred to in the Septuagint, a Greek translation of the first five books of the Hebrew bible, made in Ptolomeic Egypt in the third century BCE. A beet identified as *Beta maritima* is included in *De Material Medicus* of Pedanius Dioscorides written in the first century CE, and the first illustrated version of 512, known as the *Juliana Anicia Codex*, includes an image with non-spherical root. Beet is mentioned in several tractates of the Talmud, a sixth century collection of history and civil law written in Babylonia. *Beta maritima* possesses supernumerary root cambia, which facilitated selection of swollen rooted forms. The first colored illustration of swollen rooted table beet, *B. vulgaris*, can be found in the 1515–1517 frescos of Raphael Sanzio and Giovanni Martina da Udine in the Villa Farnesina in Rome. Swollen roots in Roman beet are illustrated and described in the 1587 French herbal *Historia Generalis Plantarum* of Jacques Dalechamps. Conically shaped beet roots are found in the market painting of Franz Snijders in the 17th century. Various spherical forms of beet root are found in the work of American painter James Peale in 1826. A complete array of beet root types is found in the Benary catalog of 1876. Modern, spherical beet roots were depicted in 1936 by the Russian painter Zinaida Serebriankov, 1936. Artistic and historical representations of table beet suggest that swollen rooted forms have existed during the past five centuries, but conically shaped roots were gradually replaced by spherically shaped roots during this period.

## Introduction

*Beta vulgaris* (L.) subsp. *maritima* (L.) Arcang, also known as sea beet, is recognized as the progenitor of the cultivated *Beta* crops, all of which are designated as *B. vulgaris* (L.) subsp. *vulgaris* and include sugar beet, mangel wurzel, Swiss chard, fodder beet, and table or garden beet. They are collectively referred to as the *B. vulgaris* complex. Current taxonomic treatments recognize four primary groups within the cultivated subspecies: leaf beet, garden or table beet, fodder beet, and sugar beet (Letschert et al., 1994; Ford-Lloyd, 2005; Wiersema and Leon, 2013). These crops are of substantial economic importance worldwide. More than 275 million tons of sugar beet was produced worldwide in 2019, with more than 30 million tons produced in the United States, supplying between 50 and 60% of United States sucrose demand (USDA-ERS, 2020). Table beet, Swiss chard, and fodder beet are important in certain regions but cultivated on a relatively small land area worldwide. Table beet is primarily consumed for its succulent root and hypocotyl, while Swiss chard is consumed for its leaves and petioles. Increasingly, table beet and Swiss chard are also important for the production of immature-leaf salad greens. Fodder beet is strictly used for livestock feed, and both mangel wurzel and fodder beet have conical, bulky roots.

Wild forms of *Beta* crops including *Beta maritima* possessed very slender roots which were not generally consumed as vegetables. During the more than 2,000 year history of *Beta* crops, humans selected swollen rooted forms that were eventually cultivated as livestock feed, sources of sucrose, and vegetables. In the process, root morphology was transformed. The objective of this paper is to examine the history and iconography of rooted forms of the *B. vulgaris* complex under domestication, emphasizing the table beet.

## Historical Background

### Botanical and Horticultural Origins

**FIGURE 1:**
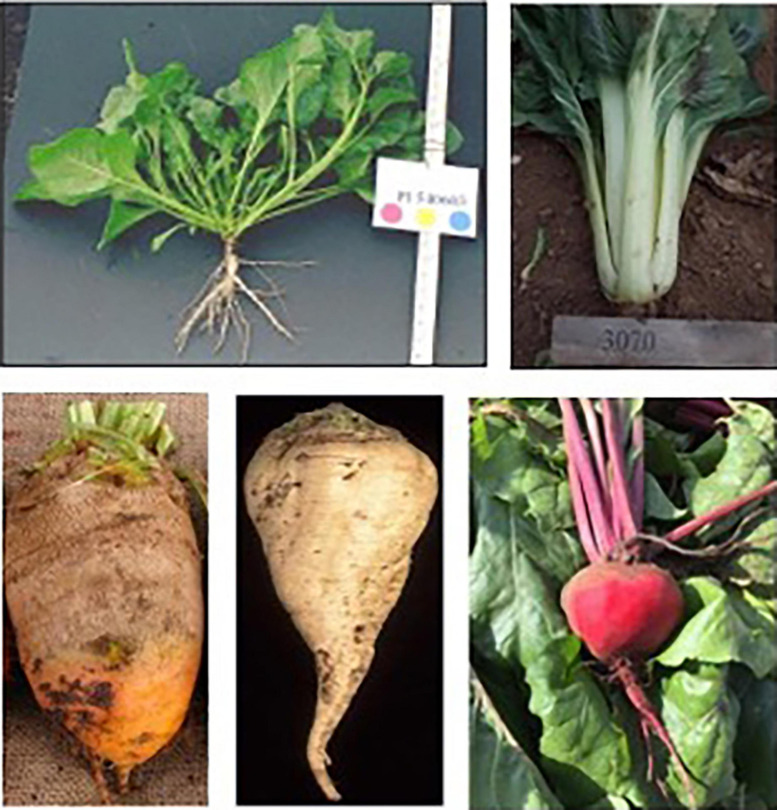
Root forms in the *Beta vulgaris* complex: (top left) wild *Beta maritima*; (top right) Swiss chard; (lower left) mangel; (lower center) sugar beet; (lower right) table beet. Sources: Lee Panella and Mitch McGrath, National Plant Germplasm System, Peggy Greb, USDA ARS, Irwin Goldman.

*Beta vulgaris* subsp. *maritima*, the progenitor of the *B. vulgaris* complex, is found along the Mediterranean coast as well as the Atlantic coast into Scandinavia, throughout the Middle East, and in India, Iran, and Azerbaijan (De Candolle, 1882; Nottingham, 2004). Roots are unswollen and often forked (Figure 1). In antiquity, sea beet was primarily consumed for its leaves although the roots were used medicinally. The root of sea beet was neither spherical nor succulent and likely difficult to prepare. The first century Roman Naturalist Pliny described the roots of beet as less fleshy than those of the saffron crocus, indicating some root swelling (Dalby, 2003). Ancient Greek, Roman, and Egyptian civilizations described the use of vegetable beet in their cuisines and medicinal remedies, but most of these recipes focused on beet leaves. These sources indicate that beet was primarily a leaf crop and not widely consumed as a root vegetable. Swiss chard, which is also a domesticated form of *B. vulgaris*, is one of the primary leafy forms of the crop available today. Table beet leaves are also widely consumed, particularly as immature leaves for salad greens (Figure 1).

**FIGURE 2:**
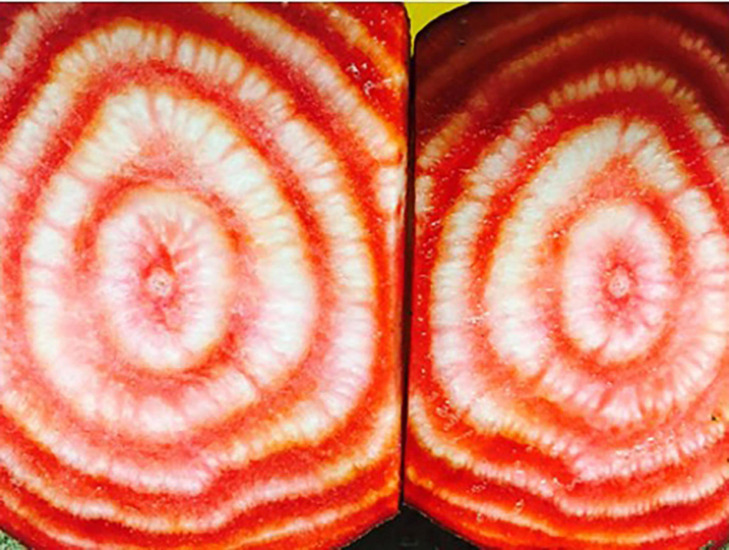
Cross section of beet with supernumerary cambial rings highlighted through differential accumulation of betalain pigments in xylem and phloem tissue. Source: Irwin Goldman (2017).

The enlarged roots of beet are possible because of the presence of supernumerary cambia which allow for root expansion into a spherical or conical form (Goldman, 2020). Supernumerary cambia exist naturally in sea beet, but the root is not particularly swollen. Artschwager (1926) indicated that supernumerary cambial layers are formed at a stage when the root is no thicker than a pencil (ca. 1 cm). The swollen nature of the modern beet root and hypocotyl is due to the simultaneous and synchronous increase in cell division and enlargement in these cambial rings (Figure 2). Milford (1973) demonstrated that 90% of the increase in girth of the root and hypocotyl was associated with the first six to eight cambial layers, even though additional cambial layers were formed during the growth of the beet plant. These cambial layers account for the visible rings in a cross-section of the roots of table beet and sugar beet, which are particularly apparent when betalain pigments accumulate at different rates in the phloem and xylem, delivering a “target” like appearance (Figure 2). The different root forms may be a function of the expression of genes controlling root expansion. Little information is available presently regarding the genetic control of beet root shape.

Domestication in *B. vulgaris* likely focused on selection for a gene trigger that expanded those cambial layers. Wild sea beet has both biennial and annual forms. Domestication of the cultivated form from wild sea beet probably focused on biennial forms, as well as those whose supernumerary cambial layers swelled to the largest degree during the first season of growth. Table beet, mangel wurzel, fodder beet, and sugar beet were all selected into their modern forms after these plants left the Mediterranean region, and most likely in Europe. The dates associated with the development of these crops are unknown, but documentary evidence for their presence does not occur until the 1500s when swollen rooted forms used for livestock feed and human use begin (Nottingham, 2004). Luigi Squalermo, in his work “*De simplicibus*” of 1561, described a beet variety from Greece called *Cochinoguglia*, with bright red, round roots like a turnip (Biancardi et al., 2012). Dalechamps (1587) later described this type as *Beta erythrorhiza*. Biancardi et al. (2012) described the writings of Pietro de Crescenzi from 1605, which documented the biennial nature of beet. His work, known as “*Ruralium commodorum*,” explicitly suggests that beet was being selected for flowering in its second season of growth following a period of vernalization, emphasizing the value of the swollen rooted vegetable for harvest at the end of the first season.

Thus, while it is impossible to pinpoint the date at which swollen rooted forms were first selected, it appears that they existed in the 16th century. Throughout this paper, we refer to spherically rooted forms as *B. vulgaris* and non-swollen rooted types as *Beta maritima*, but the long process of domestication likely transformed *Beta maritima* into an edible crop before the 16th century. The images below of beet in the sixth century from the *Julia Anicia Codex* are clearly *Beta maritima*, while the images from various sources in the 16th century are *B. vulgaris*. Transformation of *maritima* into *vulgaris* took place during this millennium but the precise details remain unclear.

Swiss chard is likely named chard because of its similarity to the French word *charde*, for leafy cardoon *Cynara cardunculus*, a plant native to the Mediterranean basin. In Italian, *bietole* and *biete* refer to both beet greens and Swiss chard. The Spanish word for Swiss chard is *acelgas*, which is derived from the Arabic word *silq*. The word “Swiss” was only applied to chard in recent times and is not associated with its early horticultural heritage. Swiss chard is likely the result of selection for leafy greens with prominent petioles, while table beet was only later selected for a swollen root.

Mangel may have originated from a cross between table beet and chard, despite the fact that chard possesses only a modest swollen root. Progeny from this cross may possess the swollen root of the table beet along with large leaves and petioles typical of chard. Mangel and fodder beet are considered the same general crop type, but mangel possesses lower dry matter content (<13%) than fodder beet. The fodder beet, which is a modern livestock food, likely originated from a cross between mangel and sugar beet (Nottingham, 2004). Among the most important cultivars of fodder beet is ‘Eckendorfer’ which was developed in the 1840s. Mangold or mangolt was the original German word for *B. vulgaris*, and mangold wurzel in German was translated as the root of the beet. Mangel is also referred to as the “root of scarcity,” and became synonymous with fodder beet. One of the most well-known characteristics of fodder beet is that a large portion of the root and hypocotyl protrudes from the soil, making hand harvest relatively easy.

Sugar beet was selected from fodder beet (McGrath and Panella, 2018). A. Margraff, a chemist, identified sucrose from *B. vulgaris* in 1747, and by 1801 a sugar factory was opened in Silesia for manufacture of sugar from white fodder beet. Fodder beet was low in sucrose concentration, perhaps only around 4% (McGrath and Panella, 2018), and thus sugar refining was inefficient. However, early efforts at scientific plant breeding were applied to increase sucrose concentration. Beet may have been one of the first crops bred using today’s modern progeny test, making use of specific gravity as a proxy for sugar concentration (Vilmorin, 1859). Within a century, sucrose concentrations more than quadrupled, and the use of sugar beet as a source of sucrose became commonplace in many of the world’s temperate regions.

Because the table beet was domesticated in Europe, we have focused our paper largely on European source material both in textual references and in artistic works. In some cases, our source material includes Middle Eastern and Asian works, as beet spread to these regions from Europe. While it appears that table beet eventually spread to many world regions outside of Europe, we have decided to focus largely on European sources from the sixth through the 19th centuries, with the addition of an early American representation from the early 19th century. The dispersion of *B. vulgaris* into India and Asia following the Age of Discovery is a subject of interest that should be explored in more detail in a separate publication.

**FIGURE 3:**
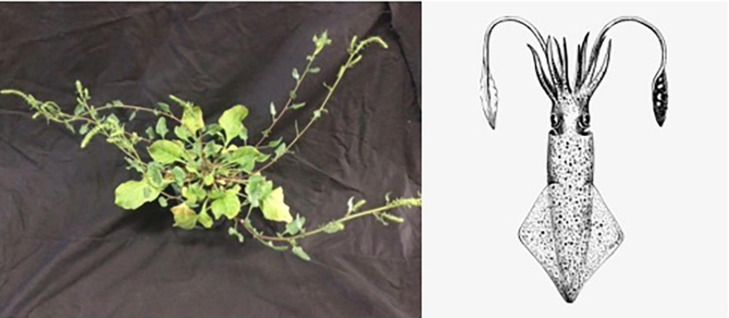
Wild sea beet, *Beta maritima* (left) and European squid, *Loligo vulgaris* (right). Source: Irwin Goldman and https://www.pngkit.com/bigpic/u2w7a9t4w7o0y3a9/.

The ancient word for beet in Greek is *teutlon*, which may refer to the cephalopod squid in the language of Ancient Greek (Figure 3). The relationship between squid and beet is unclear, though the taxonomic order for squid is Teuthida. The modern word beet comes from the Latin word *beta*, which appears to have Celtic origins. It is not entirely clear why the plant was associated with the Greek letter beta, but one hypothesis is that the seed ball, which is botanically an aggregate fruit, is reminiscent of the shape of that letter.

Beet root is referred to in the Septuagint, a Greek translation of the first five books of the Hebrew bible and the writings of the prophets, made in Ptolomeic Egypt in the third century BCE. The Septaugint was translated into Greek for Jews who lived in Egypt during the third century BCE. It was also used during the early development of Christianity as a source of the Old Testament and prophetic writings. The presence of beet in the Septaugint is, however, a mis-translation of the phrase “trapped gazelle [or antelope].” In the New King James Version of the bible, in the poetic book of Isaiah, Chapter 51, Verse 20 (Anonymous, 2021a), it is written:

Your sons have fainted, They lie at the head of all the streets, Like an antelope in a net; They are full of the fury of the LORD, The rebuke of your God.

In the Septaugint, this sentence is translated as:

Thy sons are the perplexed ones, that sleep at the top of every street as a half-boiled beet; they that are full of the anger of the Lord, caused to faint by the Lord God.

In the Hebrew bible, the phrase for the ensnared gazelle in Isaiah was actually *k’tho michmar*, which was mis-translated by the Greeks in the Septaugint as half-boiled beet. The mis-translation was perhaps due to its similarity to the Syrian word *thoreth*, which means beet, according to St. Jerome, a Latin priest who was born in the fourth century. Greek translators may have also seen the word “*mar*” in *michmar* as the Hebrew word for bitter herb. Beet may have fit the bill as a bitter herb.

Regardless of the reason for the mis-translation, the fact that the phrase “a half-boiled beet” appears in a third century BCE writing is strong evidence that the beet was consumed as a vegetable at this time. In fact, the origin of the Hebrew word for beet—*seleq*—is to “boil down.” The word *silqa* in Arabic means a well-boiled, soft vegetable, and this word was derived from *seleq*. Apparently, the condition of a boiled beet was used more widely to confer limpness; Catallus, a Latin poet from the first century, graphically equated male sexual inadequacy with the same “boiled beet” phrase (Greppin, 1990).

Beet also appears in the Talmud, a seventh century compilation of Jewish law and commentary (Anonymous, 2021b). The word *teradin* appears in tractate Eruvin, Chapter 28b and is equated by rabbinic scholars as *dor*, which means raw beet. In modern Hebrew, *tered* means spinach, but in the Talmud, scholars suggest that *tered* refers to beet. The root of the word *teradin*, *yorad*, refers to a downward direction, and is suggestive of the “boiling down” that is associated with this vegetable. The emphasis on boiling is perhaps important. In Eruvin 29a, the Talmud makes reference to the potential dangers of raw beet, suggesting that partially cooked beet is unhealthy. Rabbi Hisda, who lived in Babylonia (modern day Iraq) in the fourth century CE, wrote that a fully cooked dish of beets was beneficial for the heart, the eyes, and the intestines, and especially so when the dish sits on the stove and makes a sound as though it is continuously boiling.

Beet was well known to Greek and Romans, as well as contiguous Armenian and Arab cultures (Greppin, 1990). However, none expressed much enthusiasm for the consumption of beet root, preferring instead to use it only sparingly as a medicinal plant. They did, however, use its leaves as a vegetable, and in addition to its use in salads, dishes with fish wrapped in beet leaves were common in Greece (Greppin, 1990). A talmudic recipe from the seventh century CE included a recommendation to celebrate a festive day with a dish of beets, a large fish, and heads of garlic (Tractate Shabbat, Chapter 118b). The Roman Apicius described several beet recipes including: “slice the beets with leeks and crush coriander and cumin, add raisin wine, boil all down to perfection, bind it, serve [the beets] separate from the broth with oil and vinegar.” He also suggested cooking beets with mustard and pickling them in oil and vinegar (Apicius, 2009).

Dioscorides, a Greek physician and botanist working in the first century, described two kinds of beets: one, a white beet, which he claimed eased the bowels, and the other, a dark form, binds the bowels. He found both of them to be beneficial for earaches and for dandruff and lice. In particular, he recommended the boiled root for the treatment of pustules and burns (Beck, 2005). There is no evidence that beet possessed a swollen root in antiquity, and the first evidence of swollen roots cannot be found until the middle ages.

### Culinary Uses of Table Beet

Some of the earliest words for beet are associated with the “boiling down” of a vegetable, suggesting it was an ingredient in soup or stew, or perhaps a boiled vegetable on its own. Boiling may have been the primary method for cooking the root during this period of antiquity, when the root was not swollen and succulent. Boiled beet is referred to in the Talmud, which captures practices up through the fifth century CE. Beet root is still boiled for many recipes, though it is also likely to be roasted, steamed, or pickled. Steamed, peeled beet roots have become a popular product in Europe and the United States in recent years, as they provide a ready-to-eat product that can be consumed fresh in salads or cooked further in savory dishes. Pickled beets have been popular for many centuries, and may often have both sugar and various spices. They are typically served cold. Harvard beets, which are prepared with sugar, spices, vinegar, and butter and are served warm, can be found as a canned or jarred product in US markets. Australians and New Zealanders consume beet on sandwiches and hamburgers, much as a slice of tomato or onion would be found on such a sandwich in the United States. Beet is known as beetroot in these countries, and this term is often used in parts of Europe.

Beets possess vibrant red and yellow pigments that include the red-violet betacyanins and the yellow betaxanthins (Clement et al., 1992). Betalains are produced from the amino acid tyrosine, and Lopez-Nieves et al. (2017) suggested that relaxed sensitivity to tyrosine inhibition may explain the evolution of betalain biosynthesis. Betalains exhibit health properties such as antioxidant activity (Tesoriere et al., 2009) and the induction of Phase II enzymes that may protect against certain cancers (Lee et al., 2005). Betalains are controlled by two loci in beet, *R* and *Y* (Goldman and Austin, 2000). Beet roots are red when dominant alleles are present at these two loci, and roots are white when alleles at the *Y* locus are recessive. When the *R* locus is homozygous recessive but alleles at *Y* are dominant, the root is yellow (Hatlestad et al., 2012). Red beets typically have five to eight times more betalains than yellow beets because the conversion of tyrosine to betacyanin is far more efficient than that for betaxanthin (Wang et al., 2017). Betalain concentration for both betacyanin and betaxanthin has been increased in table beet through recurrent selection (Gaertner and Goldman, 2005). The dried, powdered product for table beet roots is used as a commercial food dye.

Beet has long been used as a primary ingredient for soup, and borscht is the name of the most well-known example of beet soup. Lee (2018) has suggested that a soup known as borscht may have originated in Ukraine sometime between the fifth and ninth centuries CE, where it was originally made from cow parsnip, *Heracleum sphondylium*, a common wild plant from the Apiaceae family found in Central Asia and Eastern Europe. Foliage from this plant was fermented and cooked with meat broth, egg, and cream to make a tart soup. This was undoubtedly a peasant food, consumed throughout Eastern Europe by the 15th century. Lee argues that by the 17th century, economic decline and social upheaval caused new ingredients to be introduced to this soup. The Polish-Lithuanian commonwealth included recipes for white borscht, which was made from fermented grain, a green borscht from sorrel or possibly chard leaves, and kvass from rye bread. Fermented cabbage was also introduced. Beet was not used as an ingredient in borscht until possibly the 16th century, when more succulent, swollen-rooted beets became available. Lee suggests that beetroot borscht was regularly made by ethnic Ukrainians east of the Dnieper River in the late 17th or early 18th century. Later, potatoes were added. Interestingly, borscht was adopted by Christians for certain fast days, with red borscht consumed on Christmas and white borscht consumed during Lent, and by Jews who used red borscht for Passover. Beet borscht became known as an inexpensive, peasant food in Eastern Europe, as exemplified by the Yiddish phrase *Bilig v’borscht*, which translates as “inexpensive, like borscht.”

## Iconography

### *Juliana Anicia Codex* (*Codex Vindoboensis*) of 512

**FIGURE 4:**
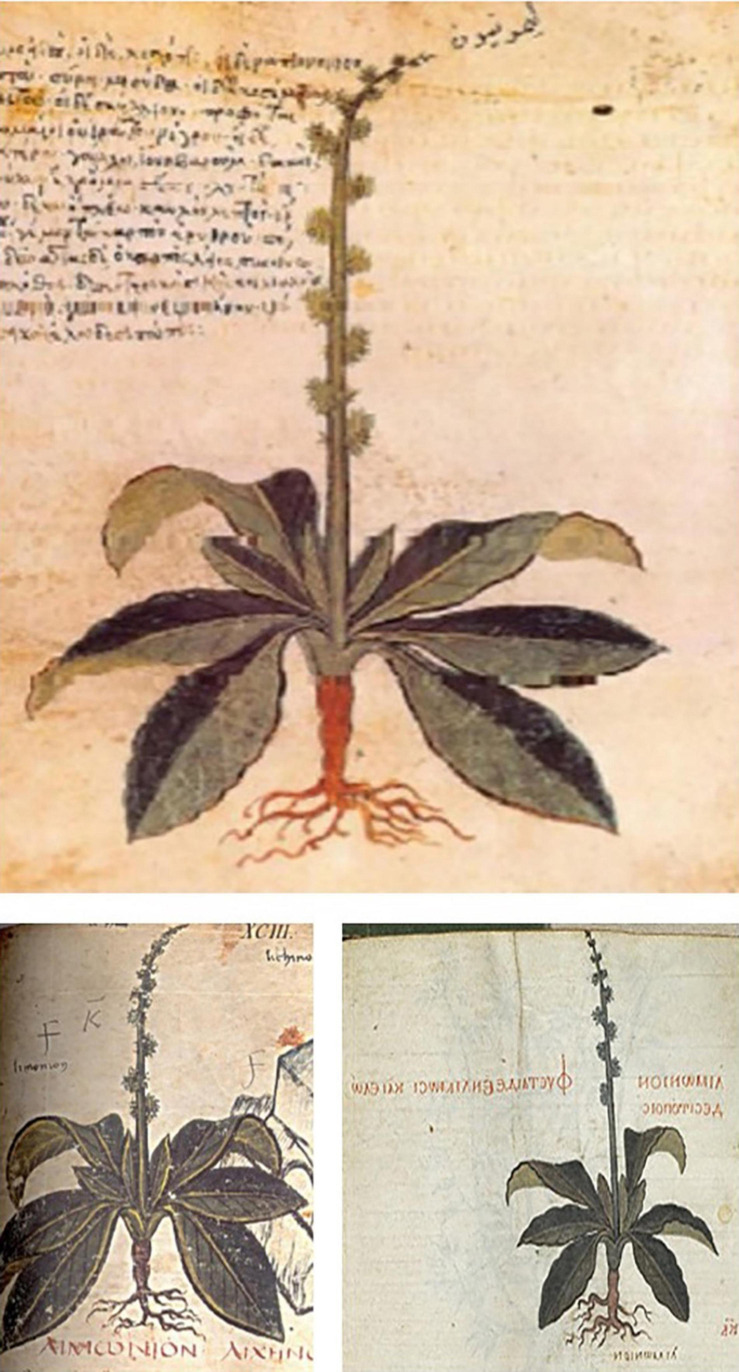
Ancient images of *Beta maritima*: (Top) Juliana Anicia Codex of 512; (Bottom left) Codex Neopolitanus, late sixth century; (Bottom right) Morgan 652, 10th century. Source: https://tulip.hort.purdue.edu/herbalimages/results.php?search=Beta+maritima.

The earliest surviving beet image can be found in the *Juliana Anicia Codex* of 512, an illustrated recension of *De Materia Medica* of Pedanius Dioscorides made in the first century CE (Figure 4). It was made as a gift to the imperial princess Juliana Anicia (462–527), daughter of Anicius Olybrius, emperor of the Western Roman Empire with its capital in Constantinople (Janick and Hummer, 2012; Janick and Stolarczyk, 2012). This volume is now the most prized possession of the Österreichische Nationalbibliothek in Vienna, Austria. The beet image has been identified as *Beta maritima* by Wellman (1958), and shows a flowering plant with rosette leaves and a reddish swollen non-spherical root (Figure 4). *Beta maritima* possesses a compressed stem, a slightly swollen hypocotyl, and a taproot. If the below-ground portions of the plant were used, they would comprise both the root and hypocotyl. An almost identical image can be found in the *Codex Neopolitanus* of about 600 (Figure 4) and the 10th century manuscript in the collection of the Morgan Library known as Morgan 6*52* (Figure 4) suggesting that these images were copied directly from the *Juliana Anicia Codex* (Janick et al., 2013). These images fit the verbal descriptions suggested by Dioscorides.

### Villa Farnesina Frescos

**FIGURE 5:**
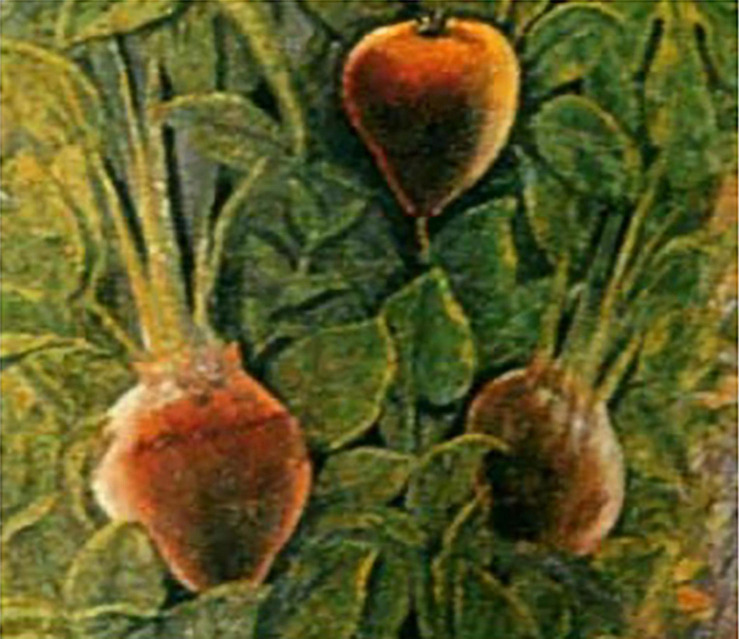
Two images of table beet with spherical roots from the festoons painted by Giovanni Martini da Udine incorporated in the Cupid and Psyche frescos of Raphael Sanzio, 1517, in the Villa Farnesina, Rome. Source: Caneva (1992), https://hort.purdue.edu/newcrop/udine/default.html.

The Roman villa of the wealthy Roman banker Agostino Chigi, now known as the Villa Farnesina, contains frescos completed in 1517 describing the heavenly adventures of Cupid and Psyche painted by Raphael Sanzo. They are surrounded by festoons containing about 170 species and 2000 images of fruits, vegetables, and flowers painted by Giovanni Martini da Udine (Caneva, 1992). The festoons included maize (*Zea mays*), pumpkins (*Cucurbita maxima*), and gourds (*Cucurbita pepo*) from seeds received from the first voyage of Columbus (Janick and Caneva, 2005; Janick and Paris, 2006). The images are realistic, often showing defects and diseases. Chigi was a humanist, and patron of poets and artists and his garden (*viridarium*) surrounding the villa was a repository of rare plants. Included among the vegetables in the festoons are the first images of beet with yellow-red spherical roots (Figure 5) described by Dalechamps in 1587 (see below).

### Sixteenth Century Herbals

#### Leonhart Fuchs

**FIGURE 6:**
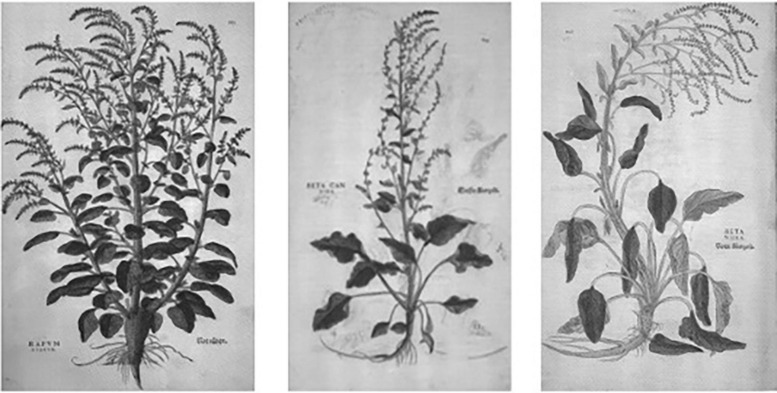
Beet images from De historia stirpium, 1552, of Leonhart Fuchs: (Left) *Beta vulgaris* subsp. *vulgaris*, Rapum Rubum; (Center) *Beta candida* (middle); *Beta nigra* (right). Source: Meyer et al. (1999).

Leonhart Fuchs’ (1501–1566) great herbal of *1542, De historia stirpium*, is well known for the first woodcut of maize. It also contains three illustrations of *B. vulgaris.* The first (Figure 6) on p. *213* titled RAVM RVBRUM = RAPUM RUBRUM is identified as *B. vulgaris* subsp. *vulgaris* (Meyer et al., 1999:363). The root is parsnip-shaped. The two other illustrations (Figure 6) on p. 806 and 807 titled BETA CANDIDA and BETA NIGRA are both identified as *B. vulgaris* subsp. *cicla* and named Spinach beet or Swiss chard (Meyer et al., 1999:558). One displays a main root that is slender, while the other root is somewhat enlarged.

#### Jacques Dalechamps

**FIGURE 7:**
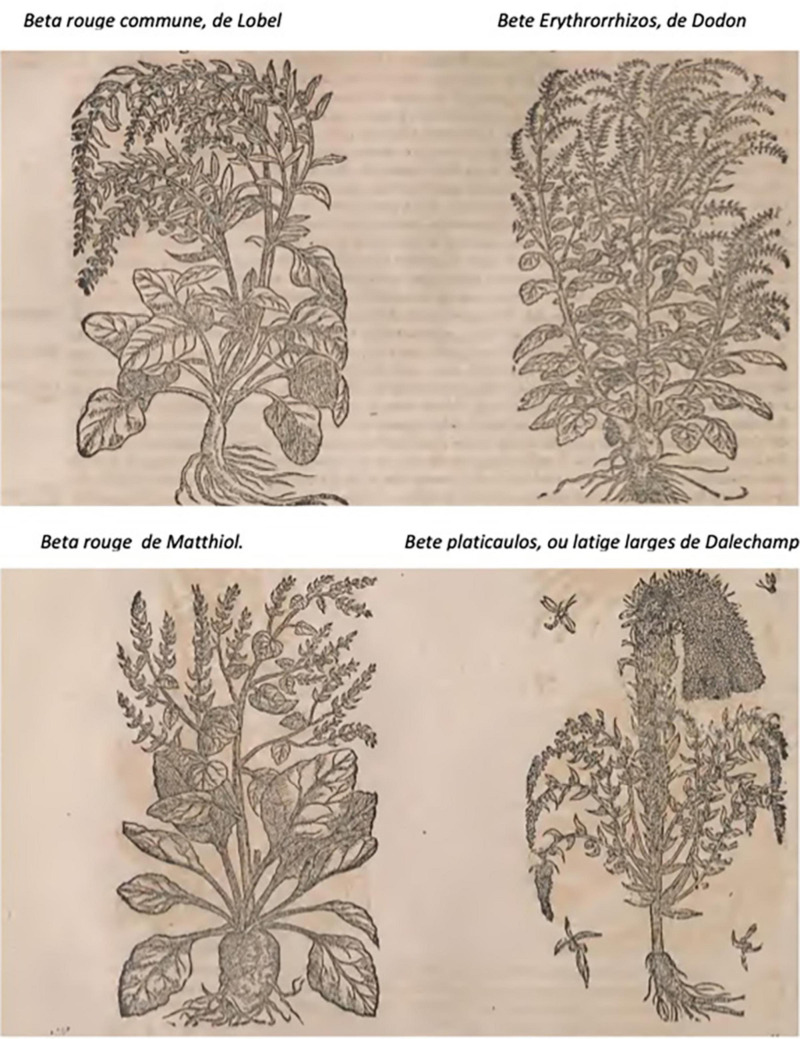
Beet illustrations from the Historia generalis plantarum of Jacques Dalechamps: (Top) white beet with swollen root (left) and black beet with spherical root (right); (Bottom) swollen spherical rooted beet (left) and slender-rooted beet with fasciated flower stalk. Source: Dalechamps (1586).

The French herbal of Jacques Dalechamps (1513–1588) published in 1586 entitled *Historia generalis deplantarum* contains beet images with both slender and spherical roots (Figure 7). The Bete rouge (red beet) on the left has a slender root in (de lobel) and a spherical root in (de matthiol).

Images from the Villa Farnesina, Leonard Fuchs, and Mattioli and Jacques Dalechamps, as well as the writings of de Cresenzi and Squalermo, indicate that swollen-rooted beet existed by the 16th century. Although there are no images to suggest that such roots existed before this time, selection for swollen-rooted forms may have taken place centuries earlier. An additional piece of information corroborating the late appearance of swollen-rooted form comes from culinary history.

#### John Gerard

**FIGURE 8:**
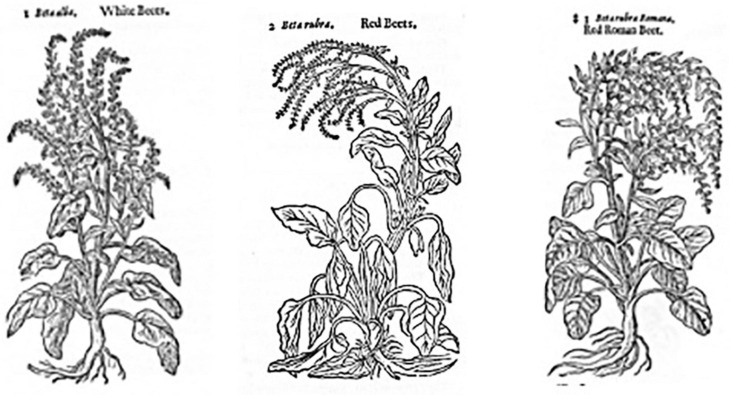
Beet images in the Herball of John Gerard. These images are referred to by the artist, from left to right, as white beet, red beet, and red Roman beet. Source: Gerarde (1597).

The beet images in the famous 1597 English herbal of John Gerard (latinized as Gerarde) show beet images not essentially different from the *Juliana Anicia Codex*. of 512 (Figure 8). Gerard’s herbal was as a translation of Rembert Dodoens’ *Stirpium Historium Pemptades Sex* of 1583 reconfigured in the arrangement of Mathias de l’Obel and Pena’s *Nova Stirpium Adversaria* of 1570 (Arber, 1938). Some of the woodcuts derived from Dodoens, but most derived from the *Eicones Plandarum seu stirpium* of Jacobus Theodorus (Tabernaemontanus). Interestingly, these images do not show swollen rooted forms.

### Seventeenth and Nineteenth Century Paintings

**FIGURE 9:**
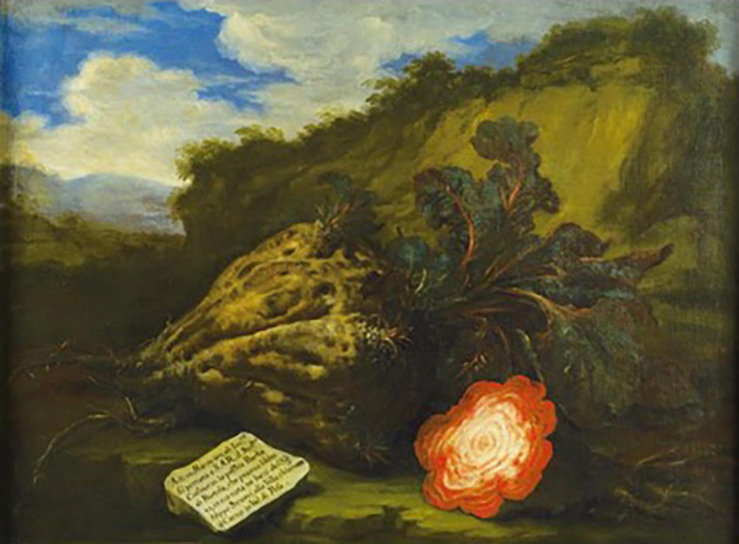
Root of beet of Filippo Strozzi, 1712, painted by Barolomeo Bimbi. Note the prominence of supernumerary cambial rings in the cut surface if this huge beet. Source: Consiglio Nazionale Delle Ricerche (1982).

Various market and still life paintings of this period show variation in the shape and size of the beet root. Bartolomeo Bimbi (1648–1729), a Florentine painter well known for his paintings of fruits and vegetable in the collections of Cosimo III, the Grand Duke of Tuscany, often included descriptions of the fruits and vegetables he painted. A painting of a giant beet (Figure 9) shows an extremely large root weighting 43 florentine pounds (14.5 kg). Such a root is likely produced from allowing a plant to grow with minimal competition among other plants and for a long growing season.

**FIGURE 10:**
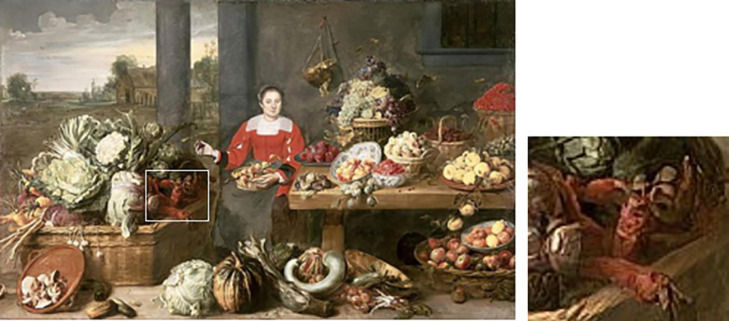
Conical beet roots in the 17th century painting “A fruit stall” by Snijders (see inset). Source: The Bowes Museum, Barnard Castle, County Durham, United Kingdom.

The market painting of Franz Snijders (1579–1657) shows red tapered or conically shaped roots that are *B. vulgaris* (Snijders, undated) (Figure 10, with detail in inset). Snijders was a Flemish painter known primarily for his still life paintings and works of market scenes and animals. He was a student of Pieter Brueghel the Younger in 1593 and had spent time in Italy before returning to Belgium to paint. Beet appears very rarely in his works, and in this case, the tapered beet is decidedly non-spherical. However, it is swollen in the crown region, exhibiting a more conical shape. Whether spherical roots were present in Belgium at this time is unknown, but evidence presented in this paper demonstrates that spherical table beet roots were present on the European continent at this time.

**FIGURE 11:**
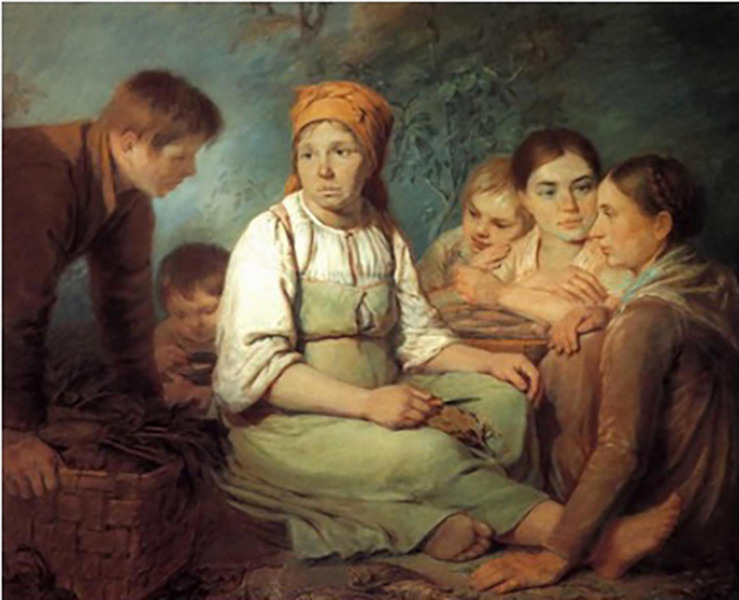
Girl peeling beet painted by Alexey Venetsianov (1820) (see insets). Source: Wikiart.org (public domain).

A similar phenotype is present in a painting “Girl shelling beet” by Alexey Venetsianov (Figure 11), a Russian painter who lived from 1780 to 1847 and specialized in depictions of peasant life. “Girl shelling beet” was painted in 1820, when Venetsianov lived in Safonovo, which is located approximately 100 km from Smolensk, about halfway between Moscow and Minsk, Belarus. In the painting, a boy leans over a wicker basket of beet roots with their tops on, and the roots are decidedly conically shaped. Their cylindrical shape is very similar to those in the Snijders painting described previously, and provides further evidence that beet roots with this elongated shape were present in rural Russia in the early part of the 19th century.

**FIGURE 12:**
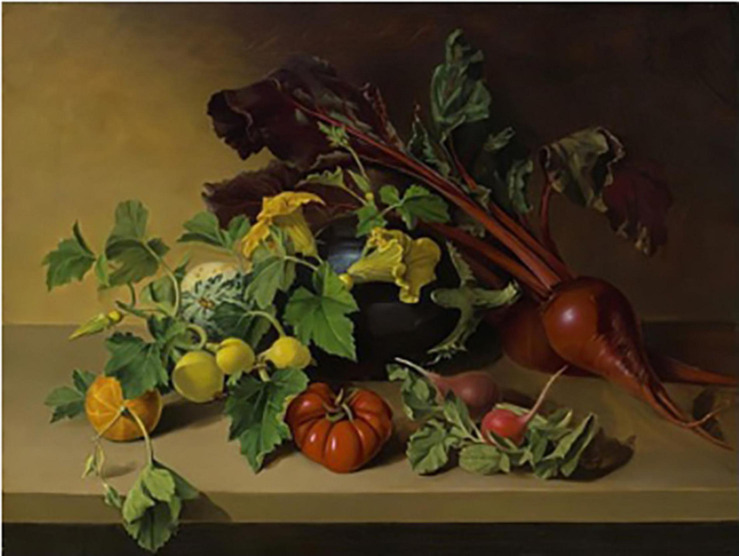
Three beets, in untitled 1826 painting of the American painter James Peale, one with foliage and a large spherical root with swollen tip and two smaller ones with spherical roots. Source: Wikimedia.com, Museum of Fine Arts, Houston, TX, United States.

An 1826 still life by the American painter James Peal (Peale, 1836) (1749–1831) shows beets with various forms of spherical roots (Figure 12). Peale, a younger brother of the well-known painted Charles Willson Peale, fought in the American Revolutionary War and was a celebrated painter of miniatures. *Still Life with Vegetables* is an extraordinary painting for its intensity of color and form. There are at least three beet roots in the painting. The largest root is tapered, but the upper half of the root is nearly identical to the modern table beet form. One of the small beets in the foreground is very similar to the modern spherical beet, while the third is slightly tapered.

The appearance of spherically rooted forms established a new market class for the table beet. While conically shaped roots may have been easier to harvest in certain soils, spherically shaped roots may have also been easy to harvest because they are often shallowly-rooted. In most cases, spherical types possess a substantial hypocotyl zone below the crown, with only the bottom third or half of the beet itself growing below the soil surface. Besides their obvious physical differences, spherically shaped beets would have likely resulted in substantially more harvestable biomass per plant than conically shaped beets. Therefore, spherical types may have resulted in increased yields of the crop. On the other hand, bulky conically shaped beets may also possess substantial yield potential, and it is entirely possible that the co-existence of these two types simply represents diversification of market classes for the crop. As is the case for many horticultural crops, there may be trends at play where a particular shape becomes common and ultimately preferred out of custom. We note that there are both conical and spherical radishes, as well as other vegetable crops, and each market class seems to have its adherents.

**FIGURE 13:**
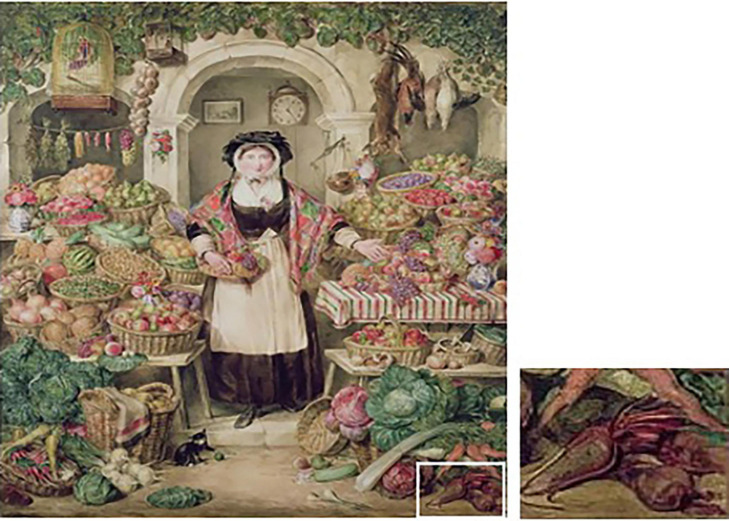
*The Vegetable Stall* by Heaphy, undated (see inset). Source: Heaphy, T.F. Undated, Source: Wikigallery.org.

“The Vegetable Stall,” (Figure 13) by the English miniaturist Thomas Frank Heaphy, depicts beet root similar to those in Peale’s painting with a larger spherical shape toward the crown of the root, and a taper toward the apex (Heaphy, undated). In Heaphy’s painting, beet root is found in the lower right portion of the painting, just beneath three orange carrots. The beet roots are perhaps more similar to those in Peal’s painting in that they have a larger spherical shape toward the crown of the root, and a taper toward the apex. Although the painting is undated, he lived from 1813 to 1873 and so it is likely that this beet root shape came to predominate during the 19th century.

**FIGURE 14:**
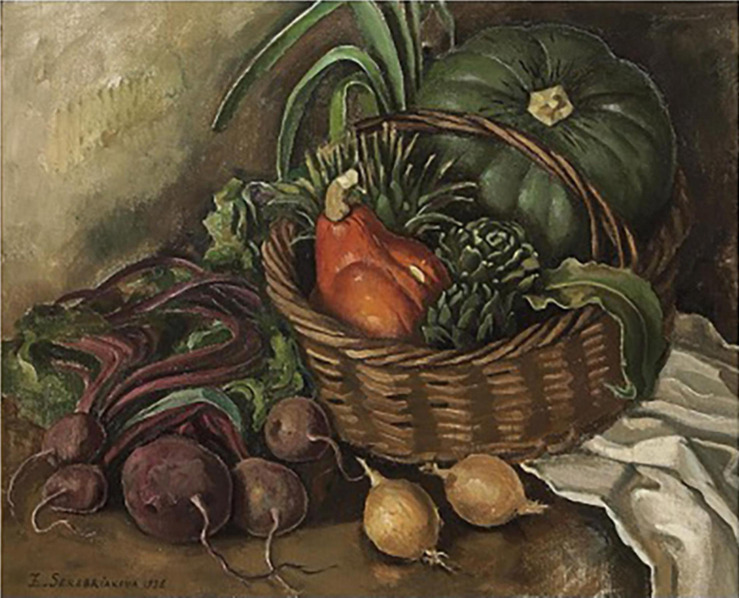
“Still life with onion, cucurbits, beetroot, and artichoke” by Zinaida Serebriakov. Source: artnet.com.

An artistic rendering of beet from 1936 by the painter Zinaida Serebriankov occurs in the painting “Still life with onion, beetroot, and artichoke” (Figure 14). Serebriakova was born in 1884 in Russia into an aristocratic family and moved to Paris in 1924. Her paintings are in the realist tradition and often display beauty in various forms. In this painting, the beet roots take a very modern shape, showing the full swollen rootedness characterized by 20th century beet roots. The red petioles display sweeping curves above the roots, echoing the rounded forms of the onion bulbs, squash fruits, and artichokes.

### Botanical Prints

**FIGURE 15:**
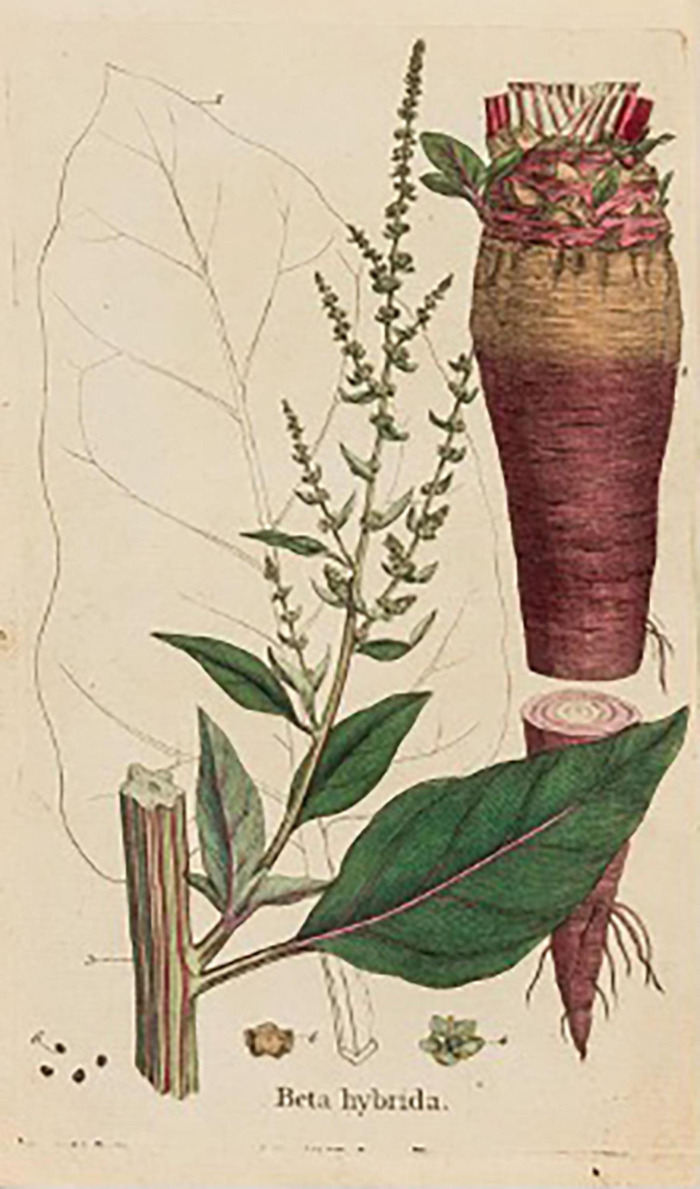
Cylindrical root of fodder beet. Source: De Commerell (1787).

*An Account of the Culture and Use of the Mangel Wurzel, or Root of Scarcity, Translated from the French of the Abbe de Commerell*, seems to have been written (or translated) by the polymath John Cloake Lettsom (1744–1815) a prolific author with an interest in fodder beet (De Commerell, 1787). Born in Tortula, British Virgin Islands, he was educated as a physician in London. The work includes a print of a fodder beet labeled *Beta hybrida* that shows a conically shaped root bisected to show its internal anatomy (Figure 15). Supernumerary cambial rings are evident.

**FIGURE 16:**
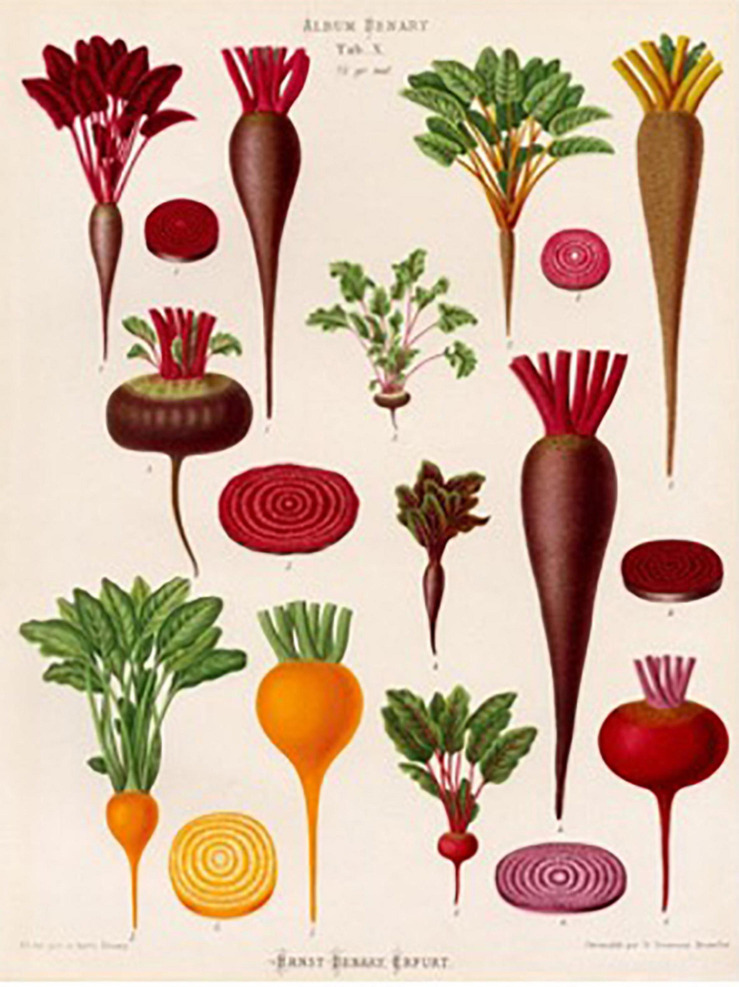
A chromolithograph plate of beet cultivars from the Album Benary: (top) the first four roots are highly tapered, conical types; (center left) flat-shaped Egyptian type; (center right) large conical root typical of a mangel; (bottom) four garden beets with spherical, orange or purple roots. Source: Benary (1876).

Ernst Benary of Jewish heritage (1819–1893) established a commercial seed and horticultural art company (Ernst Benary Samenzucht) in Erfurt, Prussia in 1843. He was largely responsible for the establishment of the German horticultural seed industry. His 1876 Album Benary contains stunning vegetable lithographs that are available as posters. The plate of beets is justly famous for displaying root morphologies (Figure 16) of different sizes, shapes, and colors.

## Conclusion

Although ancient texts prove that *B.* vulgaris was used as a medicinal since antiquity, its value as a food crop was minimal compared to its use as medicine during biblical times. It is clear that beet was used as a salad crop during this period, and that its small root was boiled and cooked prior to consumption. During biblical times, the root appears to have been of minimal significance as a food crop.

Swollen rooted forms with a more substantial, spherical root do not appear to be documented until the early 16th century. These forms were possible due to the swelling of supernumerary cambial tissues, which are responsible for the spherical shape of the beet root and hypocotyl. Swollen cambial layers must have been a selection target of farmers who were attempting to increase the size of the edible portion of the crop. Additionally, these swollen-rooted forms appeared north and northwest from the center of origin of *B. vulgaris*, as the crop moved out of the Mediterranean basin into other parts of Europe. We present iconographic evidence of spherical roots in beet in Europe as early as 1517. We are unaware of any earlier images of this shape. Subsequently, images of spherical beet roots can be found in various 16th century European herbals and later paintings. Subsequently, images of spherical beet roots may be found beginning in the 16th century in other European locations.

It is probable that spherical forms co-existed with conical or cylindrical root types for many centuries in Europe, but the paucity of spherical images suggests they were not as popular for consumption. Paintings from the 18th and 19th centuries made in different parts of Europe show conical-shaped, cylindrically shaped, and tapered beet roots in vegetable market scenes and still life paintings. Large-rooted *B. vulgaris* crops such as the mangel wurzel and sugar beet coexisted with these vegetable types. A characteristic conically shaped root typically exhibited a more spherical shape with rounded shoulders near the crown of the root, and was highly tapered. An example of this phenotype can be seen in the center right of the Benary album. The Benary album serves as evidence that the modern spherical root, as well as the complete array of root shapes currently available, was present in Europe by 1876. Twentieth century paintings begin to show the rounded, swollen-rooted beet roots typical of today’s market. Zinaida Serebriakova’s 1936 “Still life with onion, beetroot, and artichoke” shows beet in its fully modern form.

## Author Contributions

IG and JJ conceived of, researched, and wrote the manuscript equally and collaboratively. Both authors contributed to the article and approved the submitted version.

## Conflict of Interest

The authors declare that the research was conducted in the absence of any commercial or financial relationships that could be construed as a potential conflict of interest.

## Publisher’s Note

All claims expressed in this article are solely those of the authors and do not necessarily represent those of their affiliated organizations, or those of the publisher, the editors and the reviewers. Any product that may be evaluated in this article, or claim that may be made by its manufacturer, is not guaranteed or endorsed by the publisher.
